# Supplementary material for: Acetylation of histone H4 lysine 5 and 12 is required for CENP-A deposition into centromeres
Source: Nat Commun. 2016 Nov 4;7:13465. doi: 10.1038/ncomms13465 (PMC5097169; doi:10.1038/ncomms13465)
Supplement: Supplementary Information — Supplementary Figures 1-7, Supplementary Table 1 and Supplementary References [file ncomms13465-s1.pdf]

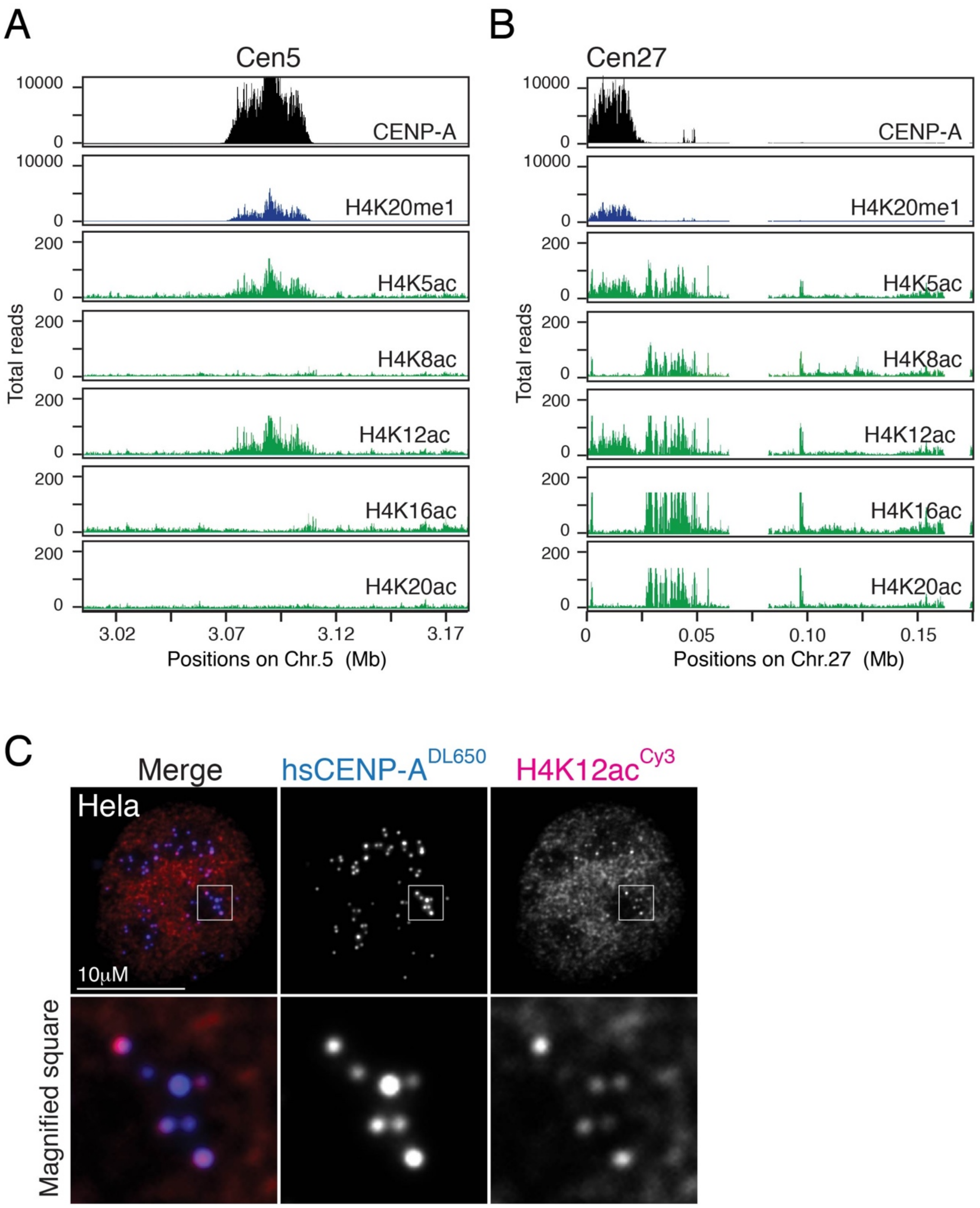

Supplementary Figure 1

Supplementary Figure 1. H4K5 and K12 acetylations are detected in centromeres

(A) High-resolution profile of ChIP-seq analysis with anti-CENP-A, anti-H4K20me1 or various antibodies against H4 modifications including K5ac, K8ac, K12ac, K16ac, and K20ac around centromere region of chromosome 5 (3.02-3.17 Mb region of chicken chromosome 5).

(B) Similar analysis of (A) around centromere region of chromosome 27 (0-0.15 Mb region of chicken chromosome 27).

(C) Immunofluorescence analysis with Cy3-labeled-anti-H4K12ac antibody (red) and DL650-labled anti-CENP-A antibody (blue) in wild-type HeLa cells. Co-localization of H4K12ac with endogenous CENP-A was observed (merge). Bar, 10 μm.

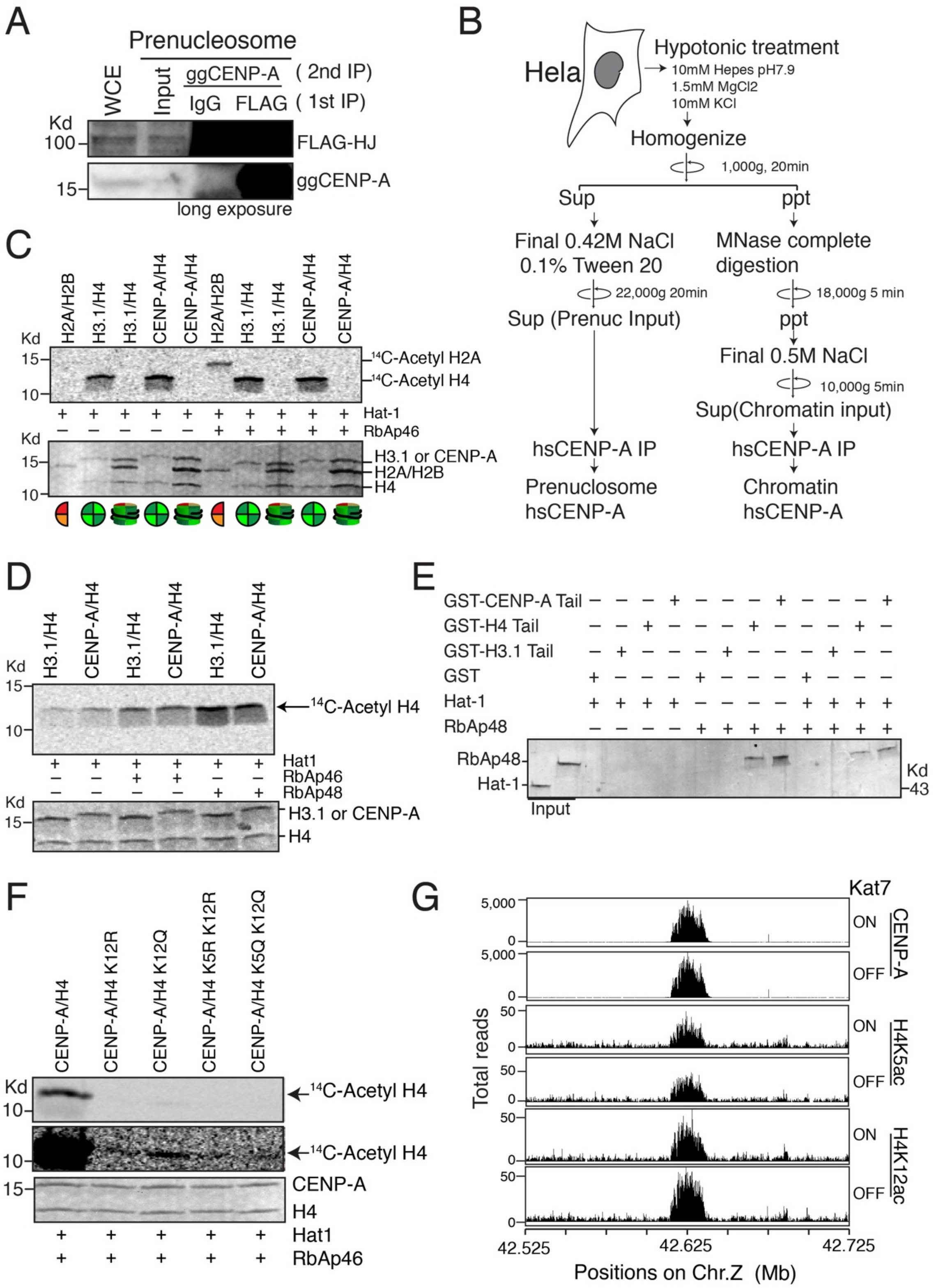

Supplementary Figure 2

Supplementary Figure 2. H4K5 and K12 acetylations primarily occurs in the pre-nucleosomal CENP-A-H4 complex

(A) Long exposure of the blot shown in Figure 2B. Both HJURP and CENP-A were detected in whole cell extract fraction.

(B) Experimental strategy for preparation of the pre-nucleosomal CENP-A-H4 complex in HeLa cells. Chromatin fraction was also prepared.

(C) The Hat1-RbAp46 complex acetylates CENP-A-H4 tetramers on histone H4. The Hat1 acetylation of histone H2A-H2B dimers (orange/red), H3.1-H4 or CENP-A-H4 tetramers (green/light green), and H3.1 or CENP-A mononucleosomes was assayed in the presence or absence of RbAp46. The top panel is an autoradiogram detecting the ^{14}C-acetylation of histone substrates and the bottom panel is a coomassie stain of the gel.

(D) The Hat1-RbAp48 complex acetylates CENP-A-H4 tetramers on H4. Histone aceylation reactions were performed with Hat1-RbAp46/48 and H3.1-H4 or CENP-A-H4 tetramers as described in (C).

(E) RbAp48 binds directly to the CENP-A N-terminal tail.

(F) The xHat1/xRbAp46 complex acetylates xCENP-A-H4 tetramers on H4K5 and H4K12. Acetylation reactions were performed in the presence of Hat1 and RbAp46. Mutation of both H4K5 and H4K12 completely eliminated detectable ^{14}C-acetylation of H4 by Hat1.

(G) High-resolution profile of ChIP-seq analysis with anti-CENP-A, anti-H4K5ac or anti-H4K12ac around centromere region of chromosome Z (42.525-42.725 Mb region of chicken chromosome Z) in either Kat7-ON or OFF cells.

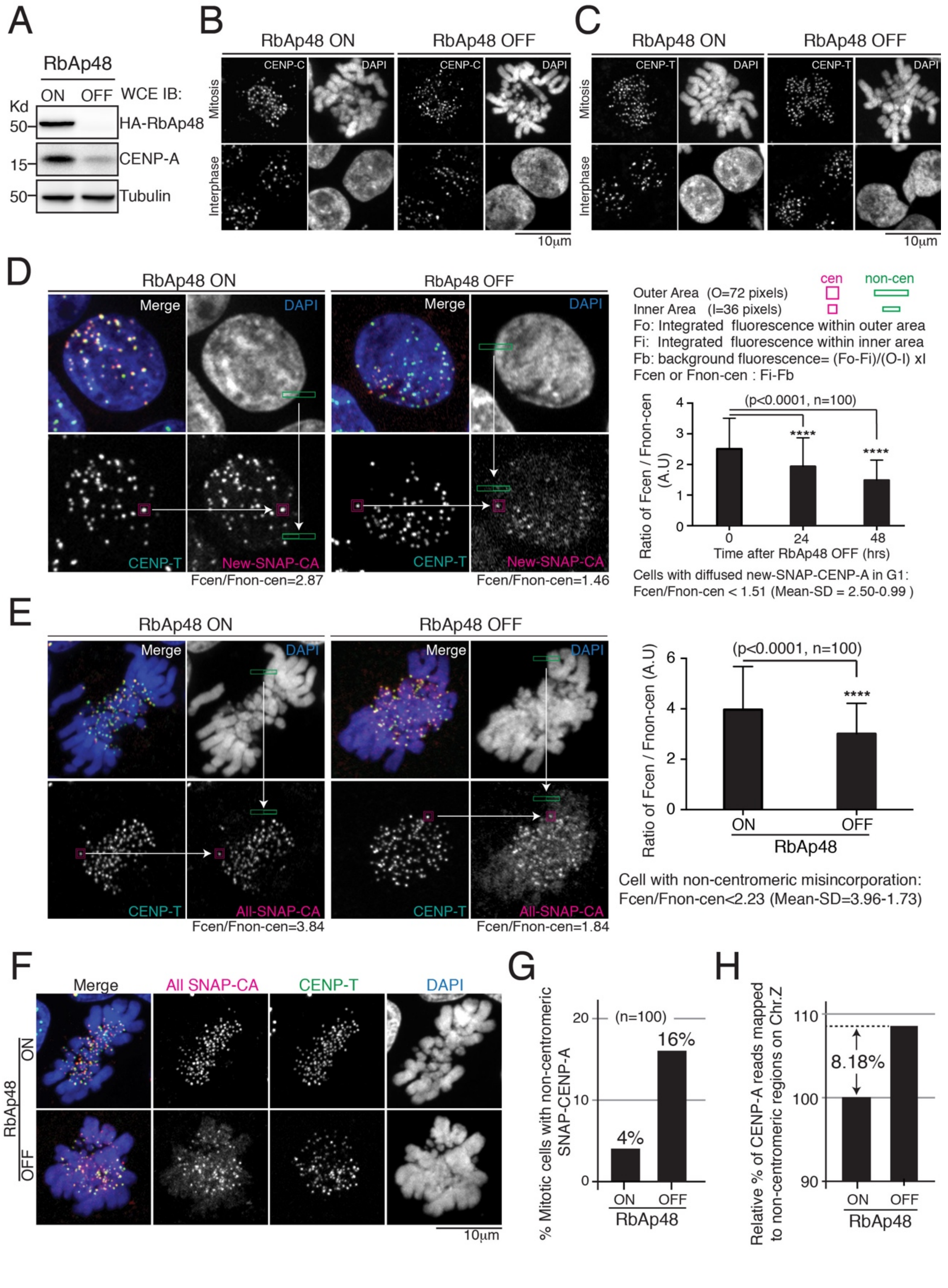

Supplementary Figure 3

Supplementary Figure 3. Phenotype of RbAp48-deficient cells

(A) Western blot analysis of whole cell extract in RbAp48-deficeint DT40 cells with anti-HA, anti-CENP-A, and anti-tubulin antibodies.

(B) Immunofluorescence with anti-CENP-C antibody in RbAp48 ON and OFF cells. Bar, 10 μm.

(C) Immunofluorescence with anti-CENP-T antibody in RbAp48 ON and OFF cells. Bar, 10 μm.

(D) Quantification method for signal intensities of centromeres and non-centromeres for SNAP-CENP-A assay used in Figure 4C. For measurement of centromere signals (SNAP-CENP-A) we followed a method described by Hoffman et al. [1] We took integrated intensities of outer area (72 pixels) and inner area (36 pixels) for each SNAP-CENP-A signal and calculated intensities according to a formula describe here. For non-centromere-region, we took integrated intensities from nucleus as inner area (36 pixels) and inner area plus adjacent non-nucleus area (36 pixels) as outer area (total 72 pixels), and then calculate intensities. Ratio of centromere signal to non-centromere-signal was plotted. When centromere signals were diffused, the ratio would be reduced. CENP-A diffused cell was defined as a cell whose centromere signals ratio to non-centromere signals is less than 1.51. Error bars represent the standard deviation (SD). Asterisk indicates statistically significance ($p<0.0001$) by Student's t-test. (N=100)

(E) Signal intensities of centromeres and non-centromeres for SNAP-CENP-A were measured. CENP-A mis-incorporated cell was defined as a cell whose centromere signals ratio to non-centromere signals is less than 2.23. Error bars represent the standard deviation (SD). Asterisk indicates statistically significance ($p<0.0001$) by Student's t-test. (N=100)

(F) Representative images of SNAP-CENP-A labeled with TMR-Star in RbAp48 ON or OFF cells without quench. CENP-T was used as a centromere marker. CENP-A mis-incorporation was observed in RbAp48 OFF cell. Bar, 10 μm.

(G) Percentages of mitotic cells that displayed CENP-A mis-incorporation. Definition of CENP-A mis-incorporation is in (E).

(H) Increase of sequence reads in non-centromere region in RbAp48 OFF cells based on ChIP-seq analysis with anti-CENP-A antibody.

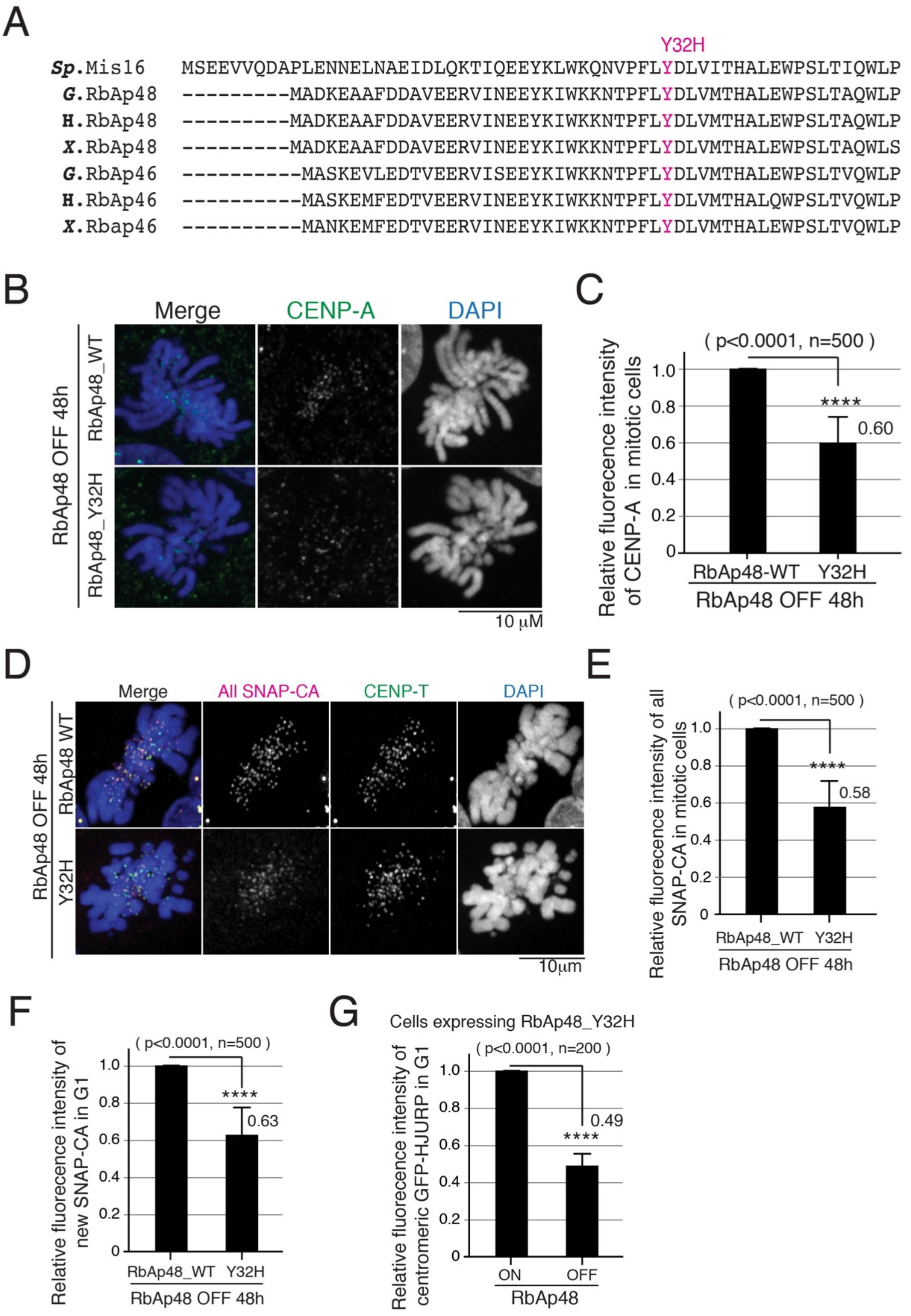

Supplementary Figure 4

Supplementary Figure 4. Phenotype of cells expressing Y32H RbAp48 mutant

(A) Multiple alignment for RbAp46/p48 sequences from various species. Conserved Y residue is highlighted. Y32H mutation was made.

(B) Representative images of CENP-A signals with anti-CENP-A antibody in RbAp48 OFF cells expressing wild-type RbAp48 or Y32H mutant RbAp48. Bar, 10 μm.

(C) Quantification of levels of CENP-A signals at kinetochores in RbAp48 OFF cells expressing either wild-type RbAp48 or Y32H mutant RbAp48. Error bars represent the standard deviation (SD). Asterisk indicates statistically significance ($p<0.0001$) by Student's t-test. (N=500)

(D) Representative images of SNAP-CENP-A labeled with TMR-Star in RbAp48 OFF cells expressing wild-type RbAp48 or Y32H mutant RbAp48. Both cell lines express SNAP-CENP-A. CENP-T was used as a centromere marker. Bar, 10 μm.

(E) Quantification of levels of SNAP-CENP-A labelled by TMR-Star at kinetochores (shown in D), in RbAp48 OFF cells expressing either wild-type RbAp48 or Y32H mutant RbAp48. Error bars represent the standard deviation (SD). Asterisk indicates statistically significance ($p<0.0001$) by Student's t-test. (N=500)

(F) Data for quench-chase-pulse experiment in RbAp48 OFF cells expressing either wild-type RbAp48 or Y32H mutant RbAp48. Both cell lines are stably expressing CENP-A-SNAP. Experimental procedure was shown in Figure 4C. Quantification of intensities by TMR-Star. Five hundred centromeres in 100 different cells were quantified for each measurement. Error bars represent the standard deviation (SD). Asterisk indicates statistically significance ($p<0.0001$) by Student's t-test. (N=500)

(G) GFP-HJURP intensities in cells expressing RbAp48 (Y32H) mutant. Endogenous RbAp48 is not expressed in OFF cells. HJURP levels were reduced in OFF cells expressing the RbAp48 (Y32H) mutant. Error bars represent the standard deviation (SD). Asterisk indicates statistically significance ($p<0.0001$) by Student's t-test. (N=200)

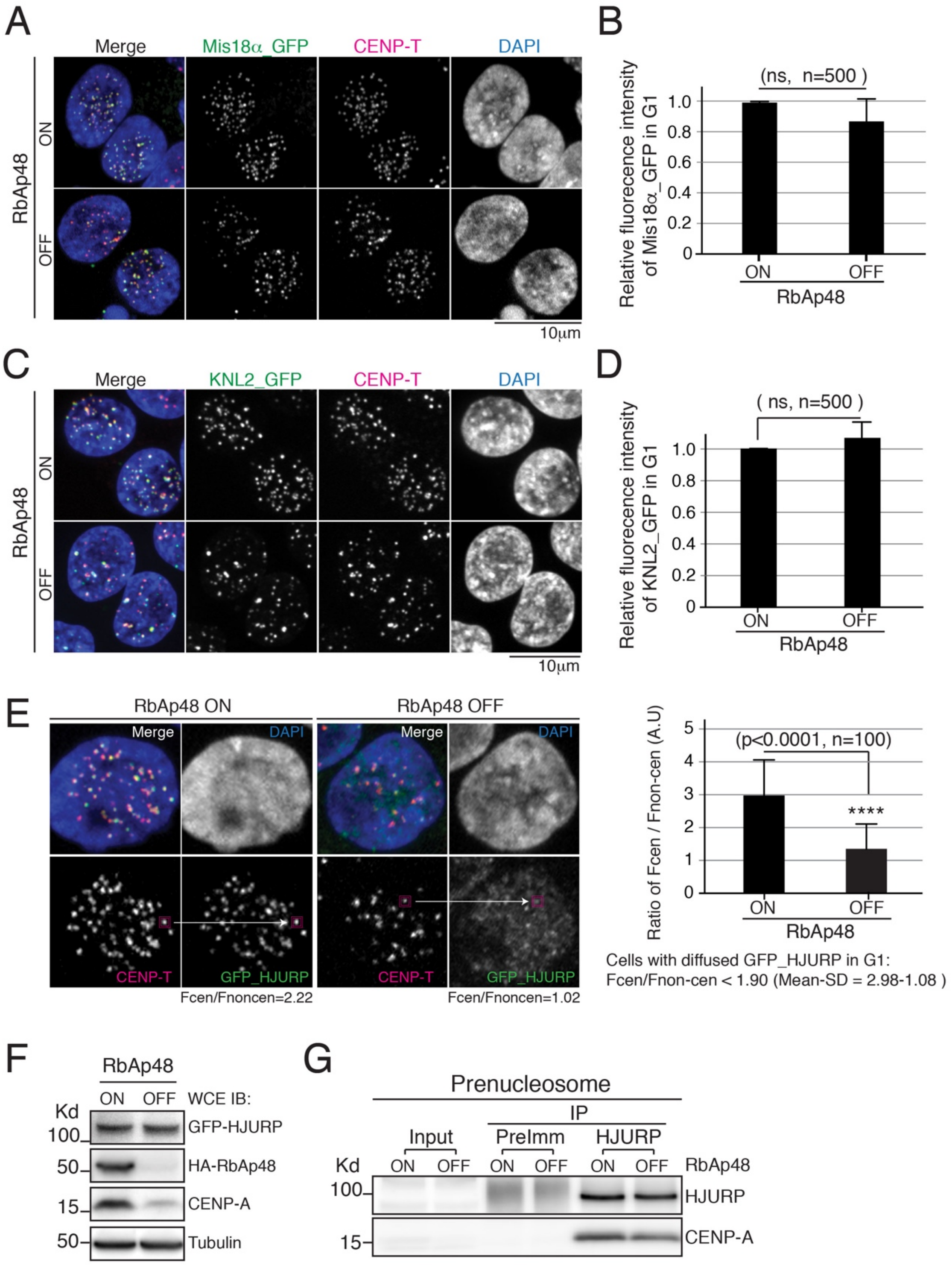

Supplementary Figure 5

Supplementary Figure 5. Phenotype of RbAp48-deficient cells

(A) Images of cells expressing Mis18α-GFP in RbAp48 ON and OFF cells. Bar, 10 μm.

(B) Relative intensities of Mis18α-GFP at centromeres in RbAp48 ON and OFF cells.

(C) Images of cells expressing KNL2(M18BP1)-GFP in RbAp48 ON and OFF cells. Bar, 10 μm.

(D) Relative intensities of KNL2(M18BP1)-GFP at centromeres in RbAp48 ON and OFF cells.

(E) Quantification for centromere and non-centromere signals for Figure 5A. HJURP diffused cell was defined as a cell whose centromere signals ratio to non-centromere signals is less than 1.90. Error bars represent the standard deviation (SD). Asterisk indicates statistically significance ($p<0.0001$) by Student's t-test. (N=100)

(F) Western blot analysis with anti-GFP, anti-HA, anti-CENP-A, and anti-tubulin antibodies in whole cell extracts in RbAp48 ON and OFF cells expressing GFP-HJURP.

(G) Western blot analysis with anti-CENP-A and -HJURP antibodies for IP samples with anti-HJURP in RbAp48 ON and OFF cells.

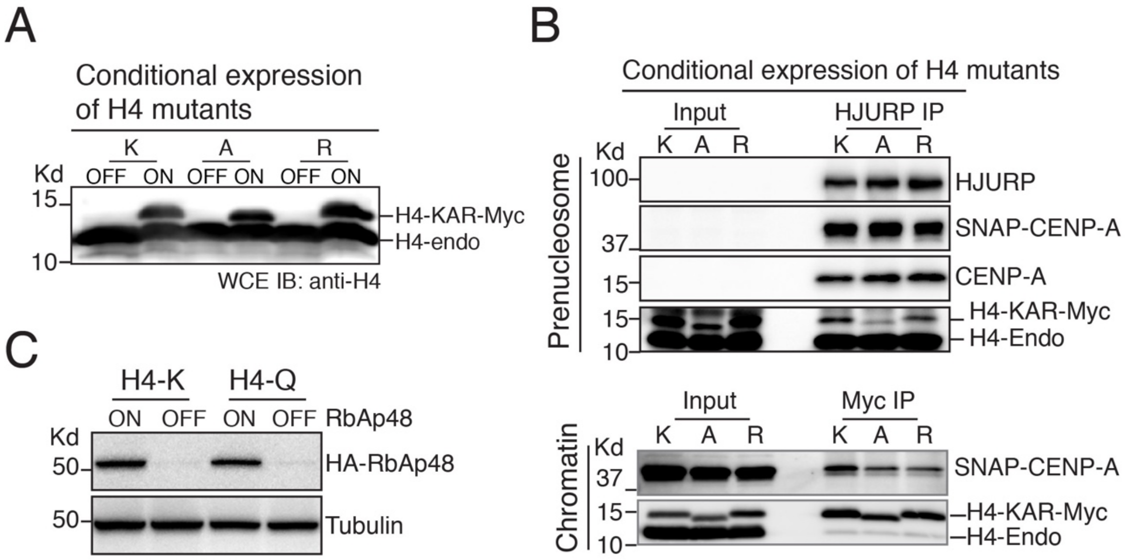

Supplementary Figure 6

Supplementary Figure 6. Conditional expression of mutant histone H4s

(A) Western blot analysis with anti-H4 antibody in cells conditionally expressing H4_K5K12 (wild-type), H4_A5A12 or H4R5R12. Expression of mutant H4s was controlled by tetracycline responsive promoter. Mutant H4s were fused with Myc tag.

(B) Immunoprecipitation with anti-HJUPR in cells expressing H4_K5K12, H4_A5A12 or H4_R5R12, followed by Western blot analysis with anti-HJURP, anti-CENP-A and anti-H4 antibodies (top). Immunoprecipitaion with anti-Myc was also performed in chromatin fraction, followed by Western blot analysis with anti-CENP-A and anti-H4 antibodies (bottom). These data indicate that mutant H4s bind to CENP-A in both pre-nucleosomal and chromatin fractions.

(C) Confirmation of suppression of RbAp48 conditional knockout cells expressing histone H4 mutants after addition of tetracycline.

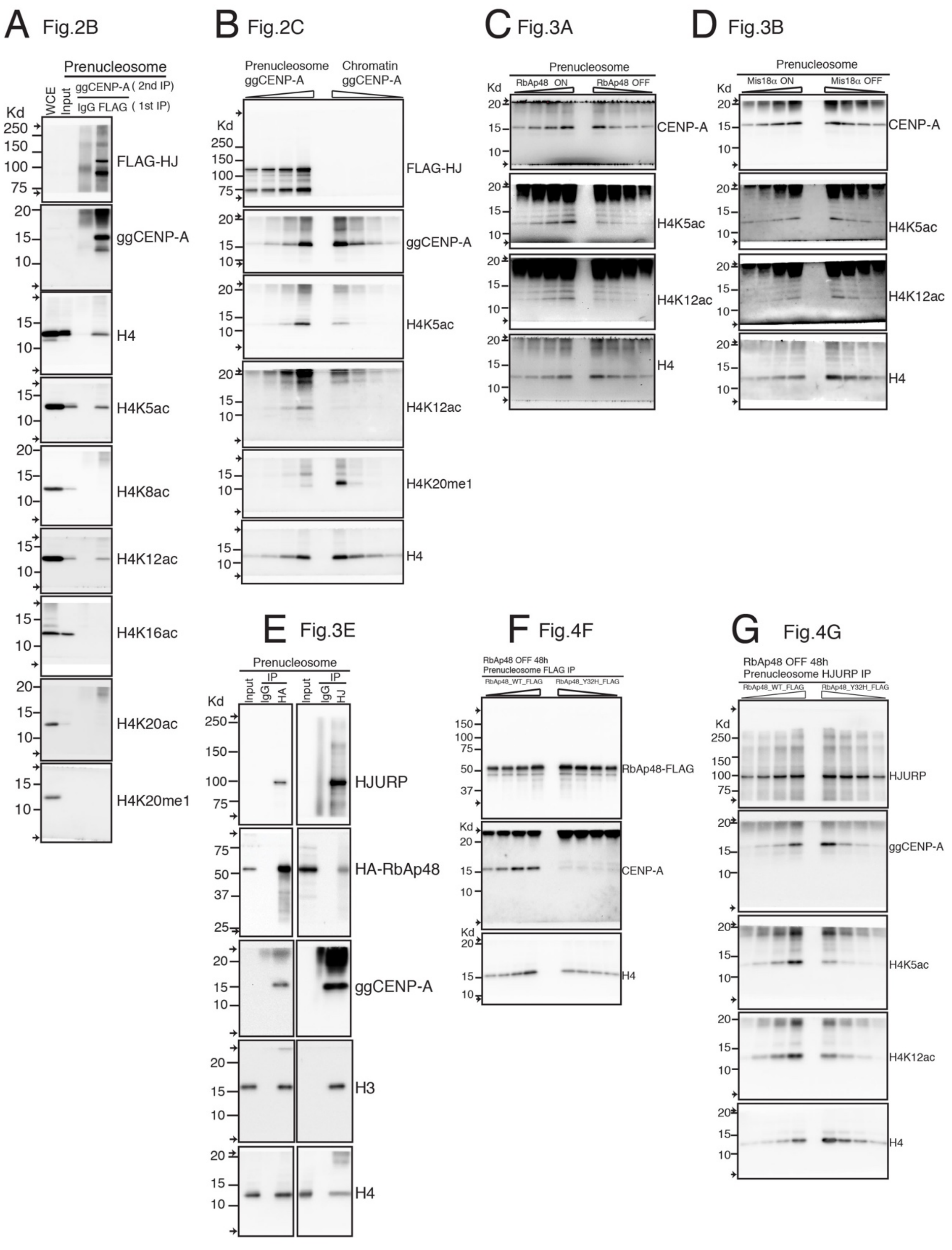

Supplementary Figure 7

Supplementary Figure 7. Uncropped blot images used in main Figures

(A) Uncropped Western blot images, which are used in Figure 2B. Arrows mean edge of membrane, which are used in each experiment.

(B) Uncropped Western blot images, which are used in Figure 2C. Arrows mean edge of membrane, which are used in each experiment.

(C) Uncropped Western blot images, which are used in Figure 3A. Arrows mean edge of membrane, which are used in each experiment.

(D) Uncropped Western blot images, which are used in Figure 3B. Arrows mean edge of membrane, which are used in each experiment.

(E) Uncropped Western blot images, which are used in Figure 3E. Arrows mean edge of membrane, which are used in each experiment.

(F) Uncropped Western blot images, which are used in Figure 4F. Arrows mean edge of membrane, which are used in each experiment.

(G) Uncropped Western blot images, which are used in Figure 4G. Arrows mean edge of membrane, which are used in each experiment.

Supplementary Table 1. A list of antibodies used in this study

Species	Antigen	Clone#	Company (Cat#)	Reference
Mouse-monoclonal	H4	200a 9C5		2
Mouse-monoclonal	H4K5ac	4A7		2
Mouse-monoclonal	H4K8ac	72A9		2
Mouse-monoclonal	H4K12ac	50B3		2
Mouse-monoclonal	H4K16ac	1B2		2
Mouse-monoclonal	H4K20ac	1B4		2
Mouse-monoclonal	H4K20me1	15F11		2
Rat-monoclonal	H3	140C-1G1		2
Mouse-monoclonal	HsCENP-A	3-19		3
Mouse-monoclonal	HA	HA-7	Sigma (H3663)	
Rat-monoclonal	HA	3F10	Roche (11867423001)	
Mouse-monoclonal	c-Myc	9E10	Wako (017-21871)	
Mouse-monoclonal	FLAG	M2	Sigma (F1804)	
Rabbit-polyclonal	ggCENP-A	serum		4
Rabbit-polyclonal	ggCENP-C	serum		5
Rabbit-polyclonal	ggCENP-T	serum		6
Rabbit-polyclonal	ggHJURP	serum		7

Supplemental References

1. Hoffman DB, Pearson CG, Yen TJ, Howell BJ, Salmon ED. Microtubule-dependent changes in assembly of microtubule motor proteins and mitotic spindle checkpoint proteins at PtK1 kinetochores. *Molecular biology of the cell* **12**, 1995-2009 (2001).
2. Hayashi-Takanaka Y*, et al*. Distribution of histone H4 modifications as revealed by a panel of specific monoclonal antibodies. *Chromosome research : an international journal on the molecular, supramolecular and evolutionary aspects of chromosome biology*, (2015).
3. Ando S, Yang H, Nozaki N, Okazaki T, Yoda K. CENP-A, -B, and -C chromatin complex that contains the I-type alpha-satellite array constitutes the prekinetochore in HeLa cells. *Molecular and cellular biology* **22**, 2229-2241 (2002).
4. Regnier V*, et al*. CENP-A is required for accurate chromosome segregation and sustained kinetochore association of BubR1. *Molecular and cellular biology* **25**,

3967-3981 (2005).

5. Fukagawa T, Pendon C, Morris J, Brown W. CENP-C is necessary but not sufficient to induce formation of a functional centromere. *The EMBO journal* **18**, 4196-4209 (1999).

6. Hori T*, et al*. CCAN makes multiple contacts with centromeric DNA to provide distinct pathways to the outer kinetochore. *Cell* **135**, 1039-1052 (2008).

7. Perpelescu M*, et al*. HJURP is involved in the expansion of centromeric chromatin. *Molecular biology of the cell* **26**, 2742-2754 (2015).
